# Magnitude of Overweight, Obesity and Insufficient Physical Sports Activities Among Secondary School Students in Kinondoni Municipal, Dar es Salaam

**DOI:** 10.24248/eahrj.v4i2.640

**Published:** 2020-11-26

**Authors:** Florence Salvatory Kalabamu, George Msengi, Namala Mkopi

**Affiliations:** a Hubert Kairuki Memorial University, Dar es salaam, Tanzania; b Muhimbili National Hospital, Dar es salaam, Tanzania

## Abstract

**Introduction:**

There is an overwhelming increase of Non-Communicable Disease worldwide such as diabetes and cardiovascular diseases. Overweight and obesity are highly associated with development of these diseases. Unhealthy lifestyle such as excessive sugar, alcohol intake and lack of adequate physical activities has been associated with development of obesity. However, these risk factors are not well elucidated among adolescents in Tanzania. We conducted this survey to determine obesity, overweight, self-reported physical activities, and preferred foods among secondary school students in Kinondoni Municipal in Dar es salaam, Tanzania.

**Methods:**

A cross sectional descriptive survey was conducted among secondary school students at Kambangwa and Makumbusho secondary schools in Kinondoni municipal in Dar es Salaam city. A simple random sampling technique was used to select participating schools with systemic random selection procedure was used to select participants. A pre structured, self-administered questionnaire was used to collect demographic information from the participants. Anthropometric measurement for Body Mass Index (BMI) was done using standard tools. Interpretation of the findings was done using World Health Organization (WHO) standard charts for age and sex. Data were analysed using Statistical Package for Social Sciences (SPSS version 20; SPSS Inc., Chicago, US).

**Results:**

A total of 234 participants were enrolled in the study. A total of 204 (87.2%) of study participants reported to regularly participate in physical sports activities. Furthermore, Males reported to participate more in physical sports activities compared to females (χ^2^ (1) =8.13., p = 0.004). During sex-wise comparison, 30 (46.2%) of males reported to participate in sports once per week compared to 71 (51.5%) of females. Reported frequency was influenced by participants’ sex (χ^2^ (3) =16.4., p= 0.001). A total of 28 (12%) participants reported fruits as their favourite food. Food preference was influenced by the participants’ sex (X 2 (5) =13.1., p < 0.02). 32(47.7%) of males reported fresh fruits juice as their favourite drink compared to 106(63.5%) of females) while 6(9%) of males reported to prefer commercial industrial juice compared to 4 (2.4%) of females

**Conclusion:**

Obesity and overweight are prevalent among secondary school adolescents in Kinondoni Municipal, Dar es salaam. In addition, the magnitude of physical activities was below the recommended amount. Therefore, awareness campaigns and advocacy programs aiming at preventive measures against NCDs such as healthy eating behaviour and promotion of physical activities among adolescents should be given high priority.

## BACKGROUND

The global burden of Non-Communicable Disease (NCD) has been increasing dramatically in recent years. From 1980 to 2014, the prevalence of hypertension and diabetes had doubled1. In 2014, The World Health Organization (WHO) estimated that the global prevalence of hypertension was 22%, 9% of these were diabetic while the global prevalence of obesity was estimated at 13%, showing to have tripled since 1975^1^. Furthermore, the same analysis showed that, around 42 million children below 5 years of age were obese. Low and middle income countries are experiencing double burden of diseases since communicable diseases such as Tuberculosis and HIV have not been well controlled, NCDs are increasing tremendously^2,3^. In 2010, the prevalence of obesity, diabetes and hypertension in Tanzania was estimated to be 5%, 7.2% and 20% respectively^4^. With the current trend, it is estimated that by 2025, the prevalence of obesity will be 8%, diabetes 9%, and 25% for Hypertension^5^. Sixty-eight percent (68%) deaths occurring worldwide are due to NCDs mainly cardiovascular diseases, diabetes and chronic lung diseases. Eighty percent (80%) of these deaths are from middle and low income countries^6,7^. Furthermore, around one-million children below 20 years of age died in 2002 as a result of NCDs^7^. In Tanzania, NCDs contribute 33% of all causes of mortality^5^.

Overall prevalence of obesity and overweight is profoundly high in developed countries compared to low and middle income countries, with Northern American countries leading with around 30% of adolescents’ population being overweight or obese followed by Europe (22%-25%) while in African countries registering between 13%-20%^8–11^. The overall prevalence in Tanzania is estimated to be around 15%. However, in Tanzania, most of the studies included pre-adolescents^12,13^. Although prevalence of obesity and overweight is relatively low in low and middle income countries compared to high income countries, the incidence rate is high due to increasing urbanisation and changes in life style^14^.

Given the current trend, it is projected that 57.3% of children will be obese at the age of 35 years^15^ while round 25% of obese adolescents will have signs of diabetes by 15 years old^16^.

Several study findings suggest that risk factors for development of NCDs start early in childhood which warrants preventive measures to be taken earlier^17,18^

Most of risk factors for NCDs are modifiable such as unhealthy diets, lack of physical activity, cigarette smoking, and excessive alcohol intake^19,20^. Usually, exposure to these risk behaviours start in early childhood and adolescence^18,21^. Over 90% of adults who smoke in United States of America started as children or youth^22^. Furthermore, heavy marketing of risky foods with high salt, fats and sugar target children and adolescents, and they are readily available especially in urban areas^23,24^. Change in children environment and technology has also led to change of lifestyle from being active to sedentary ways of living. Activities such as computer games and television watching consume children's time, attention, and prevent them from participating in physical activities^25–27^

Thus, prevention of obesity and other NCDs should start early in childhood through behaviour change strategies and promotion of healthy life style.

World Health Organization (WHO) Global Recommendations on Physical Activities for Health recommends at least 60 minutes of moderate to vigorous intensity activities daily for adolescents^28^. This includes games, sports, transportation, physical education or planned exercise context of school, family and community activities. Furthermore, it is recommended to include vegetables, fresh fruits and whole grain based carbohydrates while avoiding high fatty foods and high calorie beverages^29^. However, these healthy behaviours have been found to be low among adolescents^30–33^

Under NCD-Child support, we planned to conduct NCD advocacy program among secondary school students in Dar es salaam, but there was paucity of data on risk factors for NCDs among this age group. Therefore, we conducted this cross section survey to determine the magnitude of obesity, underweight, insufficient physical activity and food preference among secondary school children in Kinondoni Municipal in Dar es salaam. This information was collected for proper planning of the advocacy program and to provide appropriate recommendation to stakeholders after the program.

## METHODS

### Study Area

This cross sectional descriptive survey was conducted among secondary school students at Kambangwa and Makumbusho secondary schools in Kinondoni municipal in Dar es Salaam metropolitan city in June 2016. Kinondoni Municipal is one of the 5 administrative municipals located in North-West part of Dar es salaam city in Tanzania. It is occupying 321 square kilometres of land with 21 administrative wards^34^. In 2012 census, Kinondoni municipal had 929,681 inhabitants with steady population growth rate of 5% per annum and population density amounting 2,896 people per square metre. Adolescents were 186,950 which is equivalent to 22.1% of the entire population^35^

In 2018, Kinondoni had 83 secondary schools (26 public and 57 private owned) and a total of 39,295 students from form 1 to form 4^34^.

### Study Design

This cross sectional descriptive survey was conducted among adolescents in secondary schools in Kinondoni municipal with a total of 39,295 students. Kambangwa and Makumbusho secondary schools were selected by simple random sampling from a list of 65 schools obtained from the Department of Education of the municipal council in 2016 when the survey was conducted.

### Sample Size Calculation

The minimum sample size of the study participants was Calculated using Kish and Lisle formula for determination of proportion in cross-sectional studies as below:

*N=Z^2^p(1-p)/d^2^*

Where *N*=estimated sample size, *Z*=z score at 95% confidence interval (1.96), *d*=marginal error (0.05) and *p*= overall prevalence of obesity and overweight among pre-adolescents done in Dar es salaam (15%) ^13^

By using the above formula, the calculated minimum sample size was 196, but we increased the sample size by 30 in order to cover the drop out keeping in mind that it was not an invasive and risky to study participants. Therefore, 250 participants were selected. However, 234 only filled the questionnaire and presented themselves for anthropometric measurements. Those who did not turn out were not replaced.

### Ethical Considerations

The ethical clearance for conducting the survey was provided by the Ethical Review Committee of the Hubert Kairuki Memorial University with clearance REF: HK/ERC/58/06. The permission to conduct this survey and Non-Communicable Disease advocacy activity was sought from the Director of Non-Communicable Disease in the Ministry of Health, Kinondoni Municipal Executive Director and headmasters of Kambangwa and Makumbusho primary schools. We discussed with the head teachers on the aim and significance of the survey and requested for permission to discuss the same topic with students.

Written consents were sought from parents before their children (students) were enrolled in the survey. Verbal assent was sought from the participating students. Furthermore, the aim of the survey and freedom to participate or to withdraw from the survey were clearly stated in the introductory part of the questionnaire.

### Sampling Procedure

After verbal communication with form 1 up to form 4 students in schools regarding the aim, significance and risk associated with the survey, we sought their verbal assent to participate in the survey. All students who accepted were given the written consent forms in Swahili language for their parents to allow them to participate and return the filled consent form on the agreed date. For those who did not assent, and whose parents did not give consent were excluded. Systematic random sampling was used to select 250 students from 446 who met the criteria to take part in the survey. No stratification was done based on schools, age, year of study or gender.

### Data Collection

A pre-structured, self-administered questionnaire with Swahili translation was used to collect demographic information such as age, year of study and sex from the participants. Information on most favourable foods, drinks and time for physical activities were also enquired. We did not use other pretested tools for collection of physical exercise and eating habit, but we designed the questionnaire specific for our survey where self-reported information was collected. This tool was tested among few students at Makumbusho secondary school for clarity and consistency before it was used on all participants.

Standard measuring board (stadiometer) was used to measure the height of every participant and recorded in metres (m). Salter Mechanical stand on weighting scale (SECA Corporation, Humberg, Germany) was used to record the participant's weight in kilograms. The Body Mass Index (BMI) was calculated in kilograms (kg)/height (m)^2^

World Health Organization (WHO) reference charts for adolescents were used for interpretation of BMI^36,37^. These reference charts have horizontal curved lines that show the range of percentiles in relation to the BMI on the vertical axis. Those below 5^th^ percentile on the charts are considered underweight, 5^th^ to 85^th^ percentile normal, 85^th^ to 95^th^ percentile overweight and those above 95^th^ percentile are classified as obese.

### Statistical Analysis

All statistical analyses were performed using Statistical Package for Social Sciences (SPSS version 20, SPSS Inc., Chicago, USA). Continuous variables were summarised by Mean and standard Deviation. Categorical variables were summarised by frequencies and percentages. Chi Square test was used to compare frequencies in categorical variables, and p value ≤ 0.05 was considered statistically significant. Data were presented using tables and bar charts.

## RESULTS

A total 234 participants were enrolled in the survey. Females were 167 (71.4%). Participants below 15 years of age were 120 (51.3%) forming majority of participants (Table 1)

**TABLE 1:** Baseline Characteristics of Study Participants

Variable	Frequency	Percentage
**Age**		
<15	120	51.3
15–<17	88	37.6
17 –<19	21	9
≥19	5	2.1
**Total**	**234**	**100**
**Sex**		
Males	67	28.6
Females	167	71.4
**Total**	**234**	**100**
**Year of study**		
Form 1 or 2	164	70.1
Form 3 or 4	70	29.5
**Total**	**234**	**100**

65 males (97%) reported to participate in physical sports activities compared to 139 (87.3%) of females. Furthermore, 28 (16.8%) of females reported not to participate in any physical sports compared to 2(3%) of males (*χ^2^*(1) =8.13., *p*= 0.004).

During sex-wise sex comparison, 30(46.2%) of males reported to be participating in physical sports once per weeks compared to 71 (51.5%) of females (Table 2). Reported frequency was also influenced by sex of participants (*χ^2^* (3) =16.4., *p*= 0.001). 7 males (10.8%) reported to spend less than 10 minutes in each physical sports session compared to 35 (17.2%) of females while 38 (58.5%) males reported to spend more than 30 minutes per session compared to 64 (46.8%) of females (Table 2)

**TABLE 2:** Sex-Wise Comparison of BMI Interpretation of Study Participants

	Sex of Study Participants			X^2^ (df)	P value
BMI interpretation	Male N (%)	Female N (%)	Total N (%)		
less than 5th	29 (43.3)	52 (31.3)	81 (34.8)		
percentile (underweight)					
5th-85th percentile (normal)	30 (44.8)	94 (56.6)	124 (53.2)	5.6 (3)	0.13
85-95th percentile (overweight)	8 (11.9)	15 (9.1)	23 (9.9)		
above 95th percentile (obese)	0 (0.0)	5 (3.0)	5 (2.1)		
	67 (100)	116 (100)	233 (100)		

Abbreviations: X^2^ Chi-Square Test Df=Degree of Freedom BMI=Body Mass Index

In reporting favourite sports, 49 (75.4%) males reported to participate more in football while 58 (41%) of female reported netball as their most favourite physical sport. The choice of type of sports was highly influenced by participants’ sex (*χ*^2^ (4) =93., *p* < 0.001)

28 (41.8%) of males reported ugali (stiff porridge) as their favourite food compared to 47 (28.1%) females, while 36 (21.6%) of females reported to prefer French fries (chips) compared to 6(9%) males. 6 (9%) of males reported fruits among their favourite foods compared to 22(13.2%) of females forming a total of 28 (12%) participants who reported fruits as their favourite food (Table 3). The choice was influenced by the participants’ sex (*χ*^2^ (5) =13.1., *p* < 0.02). Both males and females reported fresh vegetable juice as their favourite drinks while 6(9%) of males reported to prefer commercial industrial juice compared to 4(2.4%) of females (Table 3).

**TABLE 3:** Sex-wise Comparison of Participants Involvement in Physical Sports Activities

Study Variable	Sex of Study Participants			X^2^ (df)	P value
	Male N (%)	Female N (%)	Total N (%)		
**Participants' involvement in sports activities (N=234)**					
Yes	65 (97)	139 (83.2)	204 (87.2)	8.13 (1)	004
No	2 (3)	28 (16.8)	30 (12.8)		
Total	67 (100)	167 (100)	234 (100)		
**Participants' Number of Physical Sports Activities Per Week (N=202)**					
Once	30 (46.2)	71 (51.8)	101 (50.0)		
Twice	14 (21.5)	28 (20.4)	42 (20.8)	16.4 (3)	0.001
Thrice	10 (15.4)	2 (1.5)	12 (5.9)		
More than thrice	11 (16.9)	36 (26.3)	47 (23.3)		
Total	65 (100)	137 (100)	202 (100)		
**Time Spent by Participants Per Sports Session in Minutes (N=204)**					
<10	7 (10.8)	28 (20.1)	35 (17.2)		
11-20	13 (20.0)	22 (15.8)	35 (17.2)		
21-30	7 (10.8)	24 (17.3)	31 (15.2)	5.15 (3)	0.16
>30	38 (58.5)	65 (46.8)	103 (50.5)		
	65 (100)	139 (100)	204 (100)		
**Participants' Favorite Sports (N=204)**					
Football	49 (75.4)	13 (9.4)	62 (30.4)		
Basketball	4 (6.2)	27 (19.4)	31 (15.2)	93 (4)	<0.001
Netball	4 (6.2)	58 (41.7)	62 (30.4)		
Jogging'	8 (12.3)	38 (27.3)	46 (22.5)		
Others	0 (0)	3 (2.2)	3 (1.5)		
Total	65 (100)	139 (100)	204 (100)		

Df = Degree of freedom. χ^2^ Chi-Square Test BMI=Body Mass Index

**FIGURE 1.**
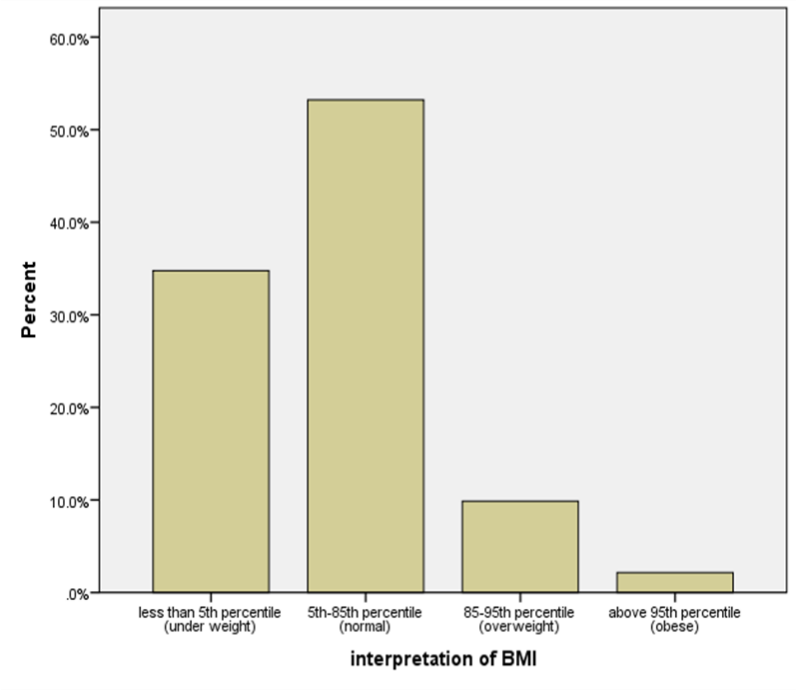
Frequency Distribution of BMI Interpretation

After taking anthropometric measurements, 23 (9.9%) of all participants were overweight while 5(2.1%) were obese (Figure 1), with 12% overall prevalence of obesity and overweight. On sex-wise comparison, 8(11.9%) of males were overweight compared to 15(9.1%) of females while all who were found to be obese were females (Table 4). However, the difference was not statistically significant (*χ*^2^ (3) =5.6., *p* < 0.13)

**TABLE 4:** Sex-wise Comparison of Participants Favorite Foods and Drinks (N=234)

Study Variable	Sex of Study Participants			X^2^ (df)	P value
	Male N (%)	Female N (%)	Total N (%)		
**Participants' Favorite Food**					
Ugali	28 (41.8)	47 (28.1)	75 (32.1)		
Rice	25 (37.3)	61 (36.5)	86 (36.8)		
French fries	6 (9.0)	36 (21.6)	42 (17.9)	13.1 (5)	0.02
Fruits	6 (9.0)	22 (13.2)	28 (12.0)		
Others	2 (3.0)	1 (0.6)	3 (1)		
Total	67 (100)	167 (100)	234 (100)		
**Favorite drink**					
Fresh fruit juice	32 (47.8)	106 (63.5)	138 (59.0)		
Soda	15 (22.4)	28 (16.8)	43 (18.4)		
Water	12 (17.9)	27 (16.2)	39 (16.7)	8.6 (4)	0.07
Commercial industrial juice	6 (9.0)	4 (2.4)	10 (4.3)		
Others	2 (3.0)	2 (1.2)	4 (1.7)		
Total	67 (100)	167 (100.0)	234 (100.0)		

Df = Degree of freedom. X^2^ Chi-Square Test

## DISCUSSION

Overweight and obesity comprised 12% of the survey participants. This is consistent with studies conducted among pre-adolescents reported by Mosha and Fungo in Dodoma and Dar es salaam^12,13^. This is also consistent with other studies conducted in s7 African countries^11^. The magnitude is relatively low compared to other developed countries. A study conducted by WHO in European region reported the prevalence of overweight and obesity among adolescents to be between 11-33% with the main predictor of obesity being from low social economic status^8–10,38^.

In our study, obesity and overweight was more prevalent in females. This is similar to other studies conducted in Tanzania^12,13^. However, in our study, the difference was not statistically significant. This difference among sexes has been attributed to hormonal changes which favour fat deposition in females and cultural restrictions of females from participating in physical sports activities^39–42^

According to our survey, 204(87.2%) of participants reported to regularly participate in physical sports activities. However, frequency and time spent during sports sessions was low compared to WHO recommendation whereby adolescents should accumulate at least 60 minutes of moderate to vigorous intensity physical activity per day^28^. Most of the physical sports should be aerobic but also strength exercise should be incorporated at least 3 times a week^28^.

Low physical activity observed in our study is consistent with other studies conducted elsewhere^43–46^. The 2012 Lancet series report estimated the global proportion of adolescents not achieving 60 minutes of Moderate To Vigorous Physical Activity (MVPA) to be to be 80.3% basing on self-reported leisure sports activities^47^.

This trend has been attributed to rapid urbanisation with increased use of modern private and public transportation such as cars, motor cycles, trains, school buses as well as other entertainments that encourage sedentary lifestyle such as computer games and television.^21,25–27,43,45^

Reported participation in physical sports was significantly lower in females (frequency per week and duration of sessions) compared to males (Table 2). This is similar to other studies conducted in both high, middle and lower income countries^48–53^. This could additionally explain the relatively higher frequency of obesity among females compared to males.

We could not quantitatively measure sedentary behaviour which is defined as time spent sitting per day in any waking activity characterised by low energy expenditure (≤1.5 metabolic equivalent) and a sitting or reclining posture due to time constrain. Sedentary behaviour includes sitting at work or school, motorized transport and screen time such as television viewing and video games^54^.

Rice and ugali (stiff porridge) were the most preferred foods while French flies (chips) were preferred by females compared to males. Only 28(12%) participants reported fresh fruits among their favourite foods which suggests lower consumption of fruits and vegetables below the recommended amount of eating 5 or more servings or 400 grams of fruits and vegetables daily^29^.

Other studies have indicated a slight increase in daily fruits and vegetable consumption, but still the amount is low compared to the recommended amount^30–32^

Fresh fruits juice and soda where the most preferred drinks. This indicates preference of sweetened and high calories foods compared to high fibre diet such as raw fresh fruits and vegetables. This trend is global due to rapid urbanisation and promotion of sweet and high sugary beverages^20,33,55–57^

## CONCLUSION

Obesity and overweight are prevalent among secondary school adolescents in Kinondoni municipal with high level of inadequate physical activities and unhealthy food preferences. Adolescents should not be sidelined in National NCDs control programs. Therefore, there is a need to establish school based health education program to provide knowledge on risk factors and consequences of NCDs, as well as to encourage them to opt for healthy eating habits and participation in physical sports activities. Schools should create supportive environment for them such as sports fields, equipment, and time.
